# Evaluation of kefir consumption on gut microbial diversity in a healthy young population using full-length 16S rRNA sequencing

**DOI:** 10.3389/fmicb.2025.1587831

**Published:** 2025-05-21

**Authors:** Yejin Choi, Gi Beom Keum, Juyoun Kang, Hyunok Doo, Jinok Kwak, Haram Kim, Yeongjae Chae, Suyoung Lee, Hyunjin Yang, Sheena Kim, Xingmin Sun, Hyeun Bum Kim, Soo Jin Yoo

**Affiliations:** ^1^Departement of Animal Biotechnology, Dankook University, Chenonan-si, Republic of Korea; ^2^Department of Molecular Medicine, Morsani College of Medicine, University of South Florida, Tampa, FL, United States; ^3^Department of Laboratory Medicine, Sanggye Paik Hospital, Inje University, Seoul, Republic of Korea; ^4^Department of Laboratory Medicine, Uijeongbu Eulji Medical Center, Eulji University, Uijeongbu, Republic of Korea

**Keywords:** kefir, short-chain fatty acids (SCFAs), 16S rRNA sequencing, probiotics, gut health, healthy young adult, gut microbiota

## Abstract

**Introduction:**

A balanced gut microbiota is essential for maintaining digestive, immune, and metabolic health. Kefir, a fermented milk beverage, influences gut microbiota through its probiotic composition and bioactive compounds, exhibiting various health-promoting effects. However, evidence on the effects of kefir on gut microbiota, particularly in healthy populations, is still limited. This study aimed to elucidate the effects of kefir on gut microbiota composition in healthy young adults under a controlled dietary environment.

**Methods:**

In this randomized, controlled, parallel-group trial, 28 healthy participants aged 18–30 years were assigned to one of three groups: kefir (*n* = 13), unfermented milk (*n* = 9), and yogurt (*n* = 6). Participants consumed 150 mL of their assigned beverage daily for 2 weeks. Stool samples were collected before and after the intervention to analyze gut microbiota composition using 16S rRNA sequencing.

**Results:**

Kefir consumption increased the relative abundance of lactate-producing bacteria, including *Bifidobacterium breve, Ruthenibacterium lactatiformans, Weissella koreensis*, and *Leuconostoc mesenteroides*. The genus *Blautia* also increased, with significant changes observed in *Blautia luti* and *Blautia wexlerae*. These shifts in species abundance were associated with increases in the short-chain fatty acid (SCFA) production pathway.

**Discussion:**

In summary, this study highlights kefir's potential to modulate gut microbiota composition in healthy individuals, emphasizing its role in supporting gut health.

## 1 Introduction

A balanced gut microbiota, known as eubiosis, plays a crucial role in maintaining overall health by promoting digestion, regulating the immune system, and synthesizing essential vitamins and nutrients. Eubiosis also contributes to metabolic homeostasis by producing short-chain fatty acids (SCFAs), which help maintain intestinal barrier integrity and reduce inflammation (Adak and Khan, 2019; Pandey et al., 2024). In contrast, dysbiosis, an imbalance in the gut microbiota, has been associated with various chronic conditions, including metabolic disorders, inflammatory diseases, and even mental health issues such as depression and anxiety. Increasing evidence suggests that the gut microbiota exerts systemic effects that extend beyond digestion, influencing immune regulation and energy metabolism (DeGruttola et al., 2016).

Among the various factors influencing gut microbiota, diet plays a primary role in modulating the composition and function of the gut microbiota. Certain foods can promote microbial diversity and maintain eubiosis, while others may contribute to dysbiosis (Singh et al., 2017). Fermented foods, in particular, are known to introduce beneficial microorganisms, such as lactic acid bacteria (LAB), and bioactive compounds that positively influence gut microbiota composition (Doo et al., 2024). In this context, there has been growing interest in functional foods containing probiotic microorganisms and bioactive compounds, particularly due to their potential to promote a healthy gut microbiome (Ganatsios et al., 2021). Reflecting this trend, the global consumption of dairy products including fermented milk beverages such as kefir has steadily increased (Prado et al., 2015; Moretti et al., 2022).

Kefir is one of the fermented foods known to exert beneficial effects on the gut microbiome. It is a milk-based beverage produced through the fermentation of milk by a symbiotic community of lactic acid bacteria, acetic acid bacteria, and yeast originating from kefir grains (Ahmed et al., 2013). Kefir has been recognized for its prebiotic properties, promoting the regulation of intestinal microbiota (Leite et al., 2015; González-Orozco et al., 2022), as well as for its ability to deliver a variety of bioactive substances generated through microbial interactions and metabolic processes during fermentation (Azizi et al., 2021; Egea et al., 2022). In addition to microbial contributions, kefir contains macro- or micronutrients such as retinol, folic acid, amino acids, and trace elements including iron, copper, zinc, and manganese, that have been shown to modulate the gut microbiota and impact host metabolism (Apalowo et al., 2024).

While a growing body of *in vitro*, animal, and clinical research supports the health-promoting properties of kefir (Bellikci-Koyu et al., 2019; Kanbak et al., 2014; Silva-Cutini et al., 2019; Liu et al., 2002; Apalowo et al., 2024), evidence specifically addressing its effects on the gut microbiota of healthy individuals remains limited, especially in comparison to more widely studied fermented dairy products such as milk and yogurt. To address this gap, the present study aimed to investigate changes in gut microbiota diversity and composition following daily kefir consumption in healthy adults, using a within-subject pre–post design.

## 2 Materials and methods

### 2.1 Subjects

Healthy volunteers aged 19–25 years were recruited from a population of Korean college students participating in a job experience camp. Only individuals who voluntarily provided written informed consent were enrolled in the study. Health status was assessed through questionnaires and interviews, which included information on hospital-diagnosed diseases, any illnesses or symptoms experienced within the past 3 months, and current health complaints. Participants were excluded from the study if they met any of the following criteria (1) use of antibiotics within the past 3 months or during the test period, (2) significant weight change within the past 3 months, (3) a diagnosis of acute or chronic systemic or gastrointestinal diseases, and (4) lactose intolerance or allergy to dairy-based foods. Participants with subjective gastrointestinal symptoms that did not meet the diagnostic criteria for a specific disease were not excluded. Participants were withdrawn from the study if they (1) failed to adhere to the scheduled consumption of the test beverage, (2) consumed other probiotic beverages (e.g., yogurt or supplements) during the study period, (3) consumed alcoholic drink during the trial period, or (4) experienced any gastrointestinal problems during the trial period.

Compliance was assessed through end-of-study interviews and review of participant-completed intake logs. Non-compliance was defined as missing the scheduled test beverage more than three times during the study period.

The study protocol was approved by the institutional review board (IRB) of Sanggye Paik Hospital (No. 2023-12-027).

### 2.2 Study design

This study was a parallel-group, randomized, controlled clinical trial. Forty participants were randomly assigned to one of three groups in a 2:1:1 ratio: a test group receiving kefir fermented milk (kefir group), a control group receiving unfermented milk (milk group), and a second control group receiving yogurt (yogurt group). Randomization was performed by a trained assistant using Microsoft Excel, and both participants and researchers were blinded to group allocation.

On the first day (day 0), all participants completed a questionnaire collecting information on demographic characteristics, health states, underlying diseases, gastrointestinal problems, dietary habits, medication use, and dietary supplements including probiotics. A one-week washout period (days 1–7) followed, during which participants were instructed to abstain from probiotic supplements. This was followed by a two-week intervention period (days 8–21). Throughout the three-week study period, all participants were provided with the same diet at the camp. During the camp, all meals were prepared in-house using fresh organic ingredients. While the diet was standardized across participants, it was neither specifically restricted nor fortified. However, from the beginning of the washout period until the end of the study, participants were prohibited from consuming any additional dairy products, fermented beverages, or probiotic-containing products other than the milk, yogurt, or kefir provided as part of the intervention.

Stool samples were collected at two time points: prior to the intervention (days 6–7) and within two days following the conclusion of the intervention (days 22–23). Along with sample collection, participants completed a follow-up questionnaire assessing subjective physical changes and their perceptions of the consumed beverages.

### 2.3 Intervention

During the two-week experimental period, participants in the kefir group consumed kefir milk, while those in the milk and yogurt groups consumed unfermented milk or yogurt, respectively. Each participant received 150 mL of their assigned beverage every morning and was instructed to consume the entire portion within 4 h.

Kefir was prepared by fermenting 1 L of sterilized, full-fat (7%) organic milk with 200 g of viable kefir grains for 24 h at 24–28°C. After fermentation, the kefir grains were separated by sieving, and the fermented milk was refrigerated at 4°C before being distributed to participants the following day. The same kefir grains were reused by adding them to fresh milk, and this process was repeated every 24 h. The kefir grains used in this study were obtained from an individual in Seoul, Korea. Microbial analysis of the kefir grains was conducted by Macrogen (Seoul, Korea). Bacterial composition, determined through 16S rRNA sequencing, revealed the presence of *Lactobacillus kefiranofaciens* (86.7%), *Lentilactobacillus kefiri* (6.9%), *Lactococcus lactis* (6.2%), and *Acetobacter fabarum* (0.1%). Fungal composition, identified through ITS gene sequencing, included *Kazakhstania unispora* (73%) and *Dekkera anomala* (27%).

The milk group received the same organic milk used for kefir preparation, without fermentation or additional processing. The yogurt group consumed a commercial yogurt product made from organic milk and fermented with *Streptococcus thermophilus* and *Lactobacillus delbrueckii subsp. bulgaricus*, without any other additives such as sugar. Commercial yogurt was selected to ensure transparency in strain composition, as manufacturers are required to disclose starter cultures. Furthermore, the standardized production process of commercial yogurt provided consistency in product quality, thereby enhancing the reliability of this study.

### 2.4 16S rRNA full-length sequence analysis

Fecal samples were collected from participants in sterile containers and stored at −70°C until analysis. Using a sterile spatula, five frozen pieces were sampled from both the surface and internal of each stool sample to obtain a total of 250 mg per sample. 250 mg of each fecal sample was placed into PowerBead Pro Plate, and DNA was extracted using the DNeasyPowerSoil Pro Kit (Qiagen, Hilden, Germany) according to the manufacturer's instructions. The extracted DNA was quantified using Quant-IT PicoGreen (Invitrogen, Waltham, MA, United States).

The sequencing libraries were prepared according to the PacBio amplicon Template Preparation and sequencing protocols to amplify the full-length 16S rRNA region (27F~1492R region). For each PCR reaction, 2 ng of genomic DNA was PCR-amplified in a 50ul reaction volume containing 10× LA PCR Buffer II (Mg2^+^-free), 2.5 mM of dNTP mix, 2.5 mM MgCl_2_, 500 nM each of the F/R PCR primer, and 5U of TaKaRa LA Taq (Takara, Kusatsu, Japan). The cycle condition for PCR was 5 min at 94°C for heat activation, and 25 cycles of 30 s at 94°C, 30 s at 53°C and 90 s at 72°C, followed by a 5-min final extension at 72°C. The primer pair with asymmetric barcoded adapters for the amplifications were as follows: 27F-F: 5′- AGRGTTYGATYMTGGCTCAG−3′, 1492-R: 5′- RGYTACCTTGTTACGACTT−3′. The PCR products were purified with SMRTbell cleanup beads. The purified product is then quantified using Quant-IT PicoGreen (Invitrogen) and qualified using the TapeStation D5000 Screen Tape (Agilent Technologies, Waldbronn, Germany). For PacBio Sequel IIe sequencing, 500 ng of pooled amplicon DNA was used for library preparation. Total 10 uL library was prepared using PacBio SMRTbell prep kit 3.0. SMRTbell templates were annealed Sequel II Bind Kit 3.1 and Int Ctrl 3.1. The Sequel II Sequencing Kit 2.0 and SMRT cells 8M Tray were used for sequencing. SMRT cells (Pacific Biosciences, Menlo Park, CA, United States) using 10 h movies were captured for each SMRT cell using the PacBio Sequel IIe (Pacific Biosciences) sequencing platform at Macrogen (Seoul, Korea).

### 2.5 Data handling and phylogenetic analysis

The raw full-length 16S rRNA sequencing data were analyzed using the Quantitative Insights into Microbial Ecology 2 (QIIME2) software package (Bolyen et al., 2019). Quality control, adapter trimming, and chimera removal were performed using the DADA2 plugin, resulting in the reconstruction of amplicon sequence variants (ASVs). The taxonomic assignment of ASVs was performed using the naïve Bayesian classifier trained on the Ribosomal Database Project (RDP) reference database (Cole et al., 2014). The multiple sequence alignment was conducted using the Multiple Alignment using Fast Fourier Transform (MAFFT) pipeline for phylogenetic diversity analysis. Alpha diversity analysis was conducted to calculate species richness and evenness using Observed features, Chao1, Shannon, and Simpson indices. Statistical differences in diversity indices between groups were evaluated using the Mann-Whitney U test. Beta diversity analysis was performed using both weighted and unweighted UniFrac distance metrics to calculate quantitative and qualitative differences in microbial community composition. Differences in microbial community structure among groups were analyzed using the analysis of similarities (ANOSIM).

### 2.6 Statistical analysis

White's non-parametric *t*-test, implemented in the Statistical Analysis of Metagenomic Profiles (STAMP) software v2.1.3, was used to identify microbial taxa with significant differences in relative abundance before and after beverage consumption (Parks et al., 2014). SparCC analysis was performed with 100 permutations to estimate pseudo *p*-values for each pairwise correlation (Friedman and Alm, 2012). The Benjamini–Hochberg false discovery rate (FDR) correction was applied to control for multiple testing, and correlations with *q* < 0.05 and |r| > 0.4 were considered statistically significant (Benjamini and Hochberg, 1995).

Functional annotation of predicted metagenomes was performed using the MetaCYC database via the PICRUSt2 pipeline (Douglas et al., 2020). Genes were classified into microbial metabolic pathways based on MetaCYC ontology to explore potential functional shifts in the gut microbiome (Caspi et al., 2016). Significant differences in the relative abundance of predicted pathways were assessed using White's non-parametric *t*-test in STAMP (v2.1.3).

Statistical analysis of demographic and health-related survey data was conducted as follows: for continuous variables, median and interquartile range (IQR) were calculated, and comparisons among the three groups were made using the Kruskal–Wallis test. For categorical variables, frequencies and percentages were reported, and group differences were evaluated using Fisher's exact test. All statistical analyses were performed using SPSS version 18.0 (SPSS Inc., Chicago, IL, USA), with a significance threshold set at *p* < 0.05.

## 3 Results

### 3.1 Subjects

A total of 40 eligible participants were randomized into three groups: 20 in the kefir group, 10 in the milk group, and 10 in the yogurt group. Eight participants (five from the kefir group and three from the yogurt group) did not begin the intervention due to recent weight change, antibiotics use, or lactose intolerance. Additionally, four participants (two from the kefir group, one from the milk group, and one from the yogurt group) discontinued the trial due to early departure from the camp or failure to provide fecal samples. Ultimately, 13 participants in the kefir group, 9 in the milk group and 6 in the yogurt group completed the two-week intervention.

Baseline characteristics of the participants were summarized in Table 1. There were no differences among the groups in terms of age, sex, height, weight, body mass index, eating habits, defection habit, abdominal symptoms, supplement use, exercise, or sleep patterns. The primary outcome of the study was the change in the relative abundance of gut microbiota in response to regular kefir consumption. Secondary outcomes included changes in gut microbial diversity and participants' subjective responses to kefir consumption.

**Table 1:** Baseline characteristics.

**Characteristics**	**Kefir group**	**Milk group**	**Yogurt group**	***p*-value**
Total N	13	9	6	
Female, N (%)	8 (61.5)	4 (44.4)	3 (50.0)	0.718
Age, median (IQR)	19.9 (19.0, 23.9)	20.5 (18.9, 22.9)	21.9 (19.9, 23.0)	0.662
Height, cm, median (IQR)	165 (159, 175)	164 (158, 175)	171.5 (162, 178)	0.628
Weight, kg, median (IQR)	65 (57, 66)	61 (53, 69)	59 (52, 74)	0.953
Body mass index, median (IQR)	22.2 (20.0, 23.9)	21.6 (20.3, 25.0)	20.5 (19.8, 23.4)	0.819
Abdominal discomfort, N (%)	7 (53.8)	6 (66.7)	1 (16.7)	0.154
Irregular defecation, N (%)	3 (23.1)	3 (33.3)	1 (16.7)	0.758
Defecation difficulty, N (%)	4 (30.8)	2 (22.2)	2 (33.3)	0.871

At baseline, the most commonly reported symptoms included abdominal discomfort or bloating (14 participants, 50.0%), irregular defecation (7 participants, 25.0%), and difficulty with defecation (8 participants, 28.6%).

At the end of the trial, no adverse effects were reported by any participants, and no one was withdrawn due to non-compliance. The post-intervention outcomes of gastrointestinal and defecation symptoms are summarized in Table 2. Post-intervention surveys revealed improvement in abdominal symptoms in nine participants overall (32.1%), including six (46.2%) in the kefir group. Improvement of defecation was reported by 11 participants overall (39.3%), and by 7 (53.8%) in the kefir group. Regarding kefir palatability, five participants (38.5%) rated the taste as good from the start, five (38.5%) reported improved perception over time, one (7.7%) responded “so-so,” and two (15.4%) rated the taste as bad.

**Table 2:** Response to intervention of participants.

**Intervention outcomes**	**Kefir group**	**Milk group**	**Yogurt group**	***p*-value**
GI symptom improvement, *N* (%)	6 (46.2)	2 (22.2)	1 (16.7)	0.327
Defecation improvement, *N* (%)	7 (53.8)	2 (22.2)	2 (33.3)	0.310

### 3.2 Microbial diversity

To assess baseline differences in microbiota composition, ANOSIM was performed on pre-intervention microbiota samples. The analysis indicated no significant differences in community structure among groups prior to the intervention (R = 0.040192, *p* = 0.26 for weighted UniFrac; R = −0.028667, *p* = 0.635 for unweighted UniFrac) (Supplementary Figure 1).

**Figure 1:**
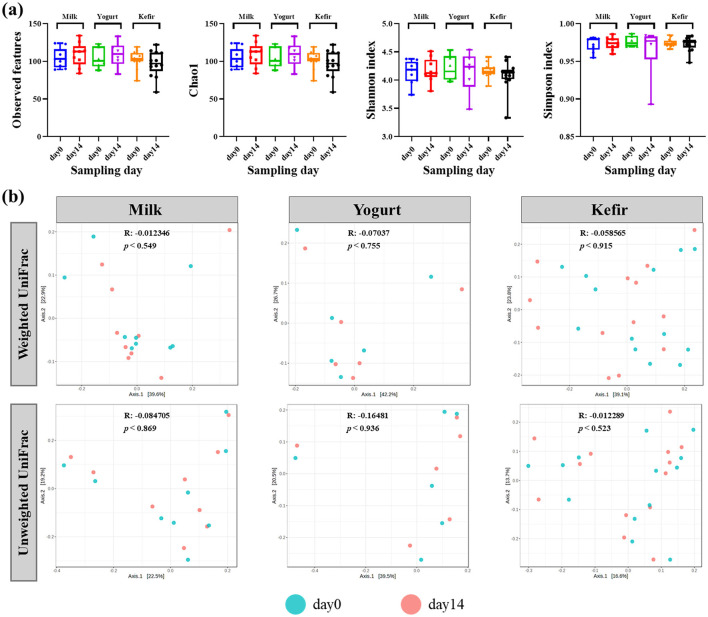
**(a)** Box plots of the alpha diversity (Observed OTUs, Chao1, Shannon index, and Simpson index) of gut microbiota before and after consuming milk, yogurt, and kefir. The plots illustrate the microbial richness and evenness in the gut microbiota. **(b)** Principal coordinates analysis (PCoA) plots based on weighted and unweighted UniFrac distances metrics. The 'R' values represent the correlation coefficients, and '*p*' values indicate the statistical significance of community differences over time.

In addition, bacterial diversity within each group was analyzed to evaluate changes before and after dietary intervention with kefir, milk, or yogurt. Alpha diversity analysis, using the number of observed OTUs, Chao1, Shannon, and Simpson indices, were employed to evaluate species richness and evenness within the microbial communities (Figure 1a).

The analyses revealed no statistically significant differences in alpha diversity before and after the intervention in any of the groups. Additionally, no significant differences in alpha diversity were observed between the groups.

Further analysis of microbial community composition using both weighted and unweighted UniFrac distances showed no significant shifts in beta diversity over the course of the intervention. Principal Coordinate Analysis (PCoA) plots indicated minimal phylogenetic variation in the microbial communities across the study period (Figure 1b).

### 3.3 Gut microbiota composition

**Figure 2:**
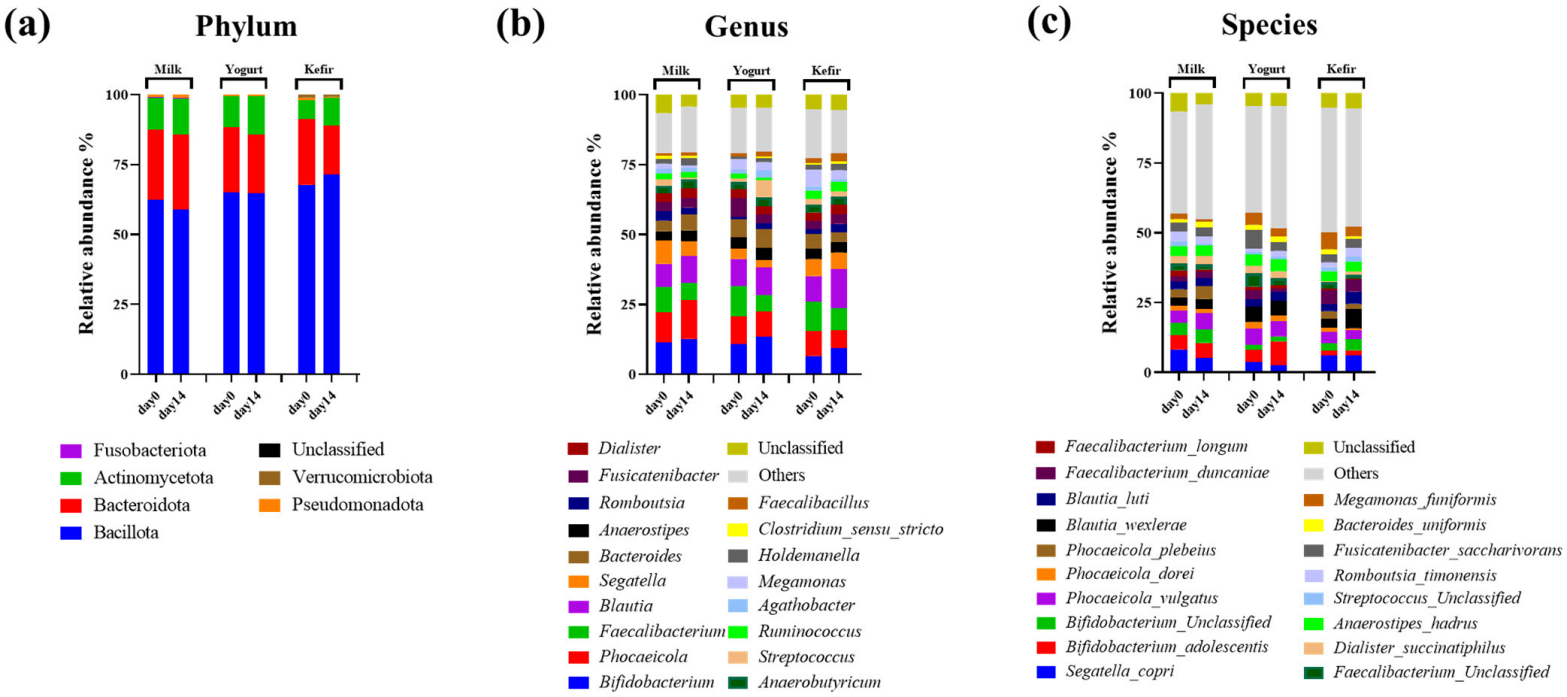
Microbial compositions of gut bacteria at the **(a)** phylum, **(b)** genus, and **(c)** species levels.

Taxonomic classification of sequences identified a total of 6 phyla, 106 genera, and 204 species, with several genera and species dominating across the samples. At the phylum level, Bacillota and Bacteroidota were predominant in all groups (Figure 2a). In the milk group, the relative abundance of Bacillota decreased from 62.38% to 58.98%, while Bacteroidota increased from 25.04% to 26.59%. In the yogurt group, Bacillota remained stable at 65%, while Bacteroidota declined from 23.42% to 20.88%. The kefir group showed an increase in Bacillota from 67.73% to 71.46%, and a decrease in Bacteroidota from 23.45% to 17.32%.

At the genus level, the relative abundance of the genus *Bifidobacterium* within the Actinobacteria phylum increased in all groups: from 11.54% to 13.03% in the milk group, 11.10%−13.76% in the yogurt group, and 6.87%−9.92% in the kefir group. The genus *Blautia* also showed changes in relative abundance, rising in the milk group (8.44%−9.64%) and the kefir group (9.17%−13.98%), while remaining stable in the yogurt group (9.76%−9.99%) following the intervention (Figure 2b).

At the species level, within the genus *Blautia, Blautia wexlerae* increased from 3.22% to 6.76%, and *Blautia luti* increased from 2.60% to 4.32% in the kefir group. The change in *Blautia luti* was statistically significant (*p* < 0.05) (Figure 2c).

**Figure 3:**
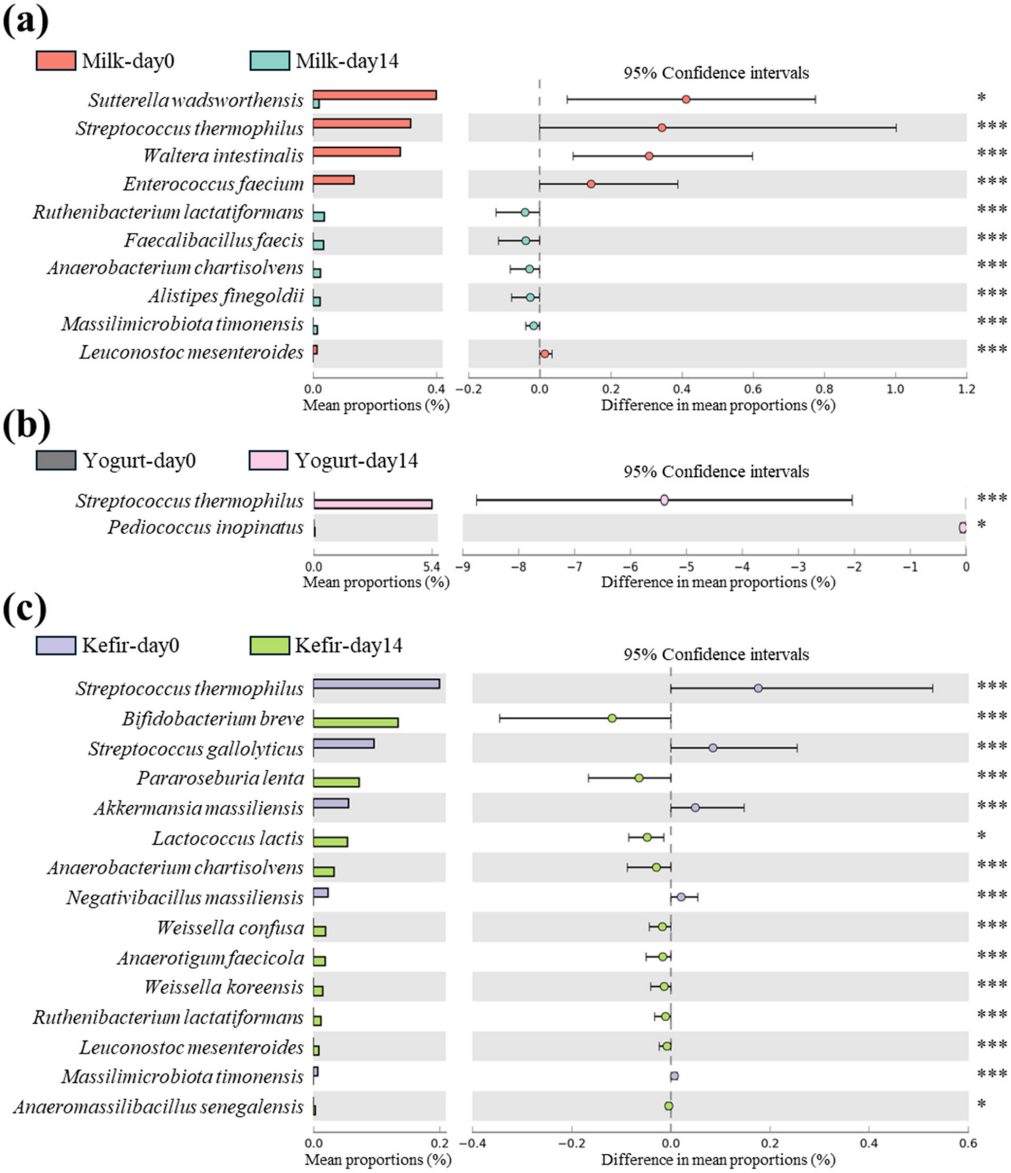
Differential abundance of species in gut microbiota following consumption of milk, yogurt, and kefir over time. Extended error bar plot indicates the significant changes in the abundance of specific microbial species in the gut microbiota of subjects before and after consuming **(a)** milk, **(b)** yogurt, and **(c)** kefir. Species showing statistically significant differences in their proportions are analyzed using White's non-parametric *t*-test, with the results depicted as bar graphs. Each bar represents the mean proportion of a species, and the lines indicate the 95% confidence intervals. Significant changes are denoted by asterisks: *p < 0.05, ***p < 0.001.

In the milk group, *Streptococcus thermophilus* showed a significant decrease after the intervention (*p* < 0.001) (Figure 3a). In contrast, the yogurt group, where *S. thermophilus* was used as the fermentation starter, showed a significant increase in this species after 2 weeks of consumption, with no notable changes in other species (*p* < 0.001) (Figure 3b). In the kefir groups, *S. thermophilus* showed a significant decrease similar to the milk group after the intervention (*p* < 0.001) (Figure 3c).

In the kefir group, there was a significant increase in *Lactococcus lactis*, one of the species that makes up kefir grains (*p* < 0.05). Additionally, the relative abundances of several lactose-fermenting species, including *Bifidobacterium breve, Ruthenibacterium lactatiformans, Weissella koreensis*, and *Leuconostoc mesenteroides* were significantly increased after the intervention (*p* < 0.001) (Figure 3c).

Detailed quantitative data on microbial taxa were provided in Supplementary Table 1.

### 3.4 Core microbiome

The core microbiome refers to a group of microbial species that consistently occupy the gut ecosystem and maintain stable relative abundance across samples. These species are essential for maintaining the stability and function of the gut ecosystem, supporting critical processes such as immune regulation, digestion, and nutrient absorption (Zhao, 2025; Risely, 2020).

**Figure 4:**
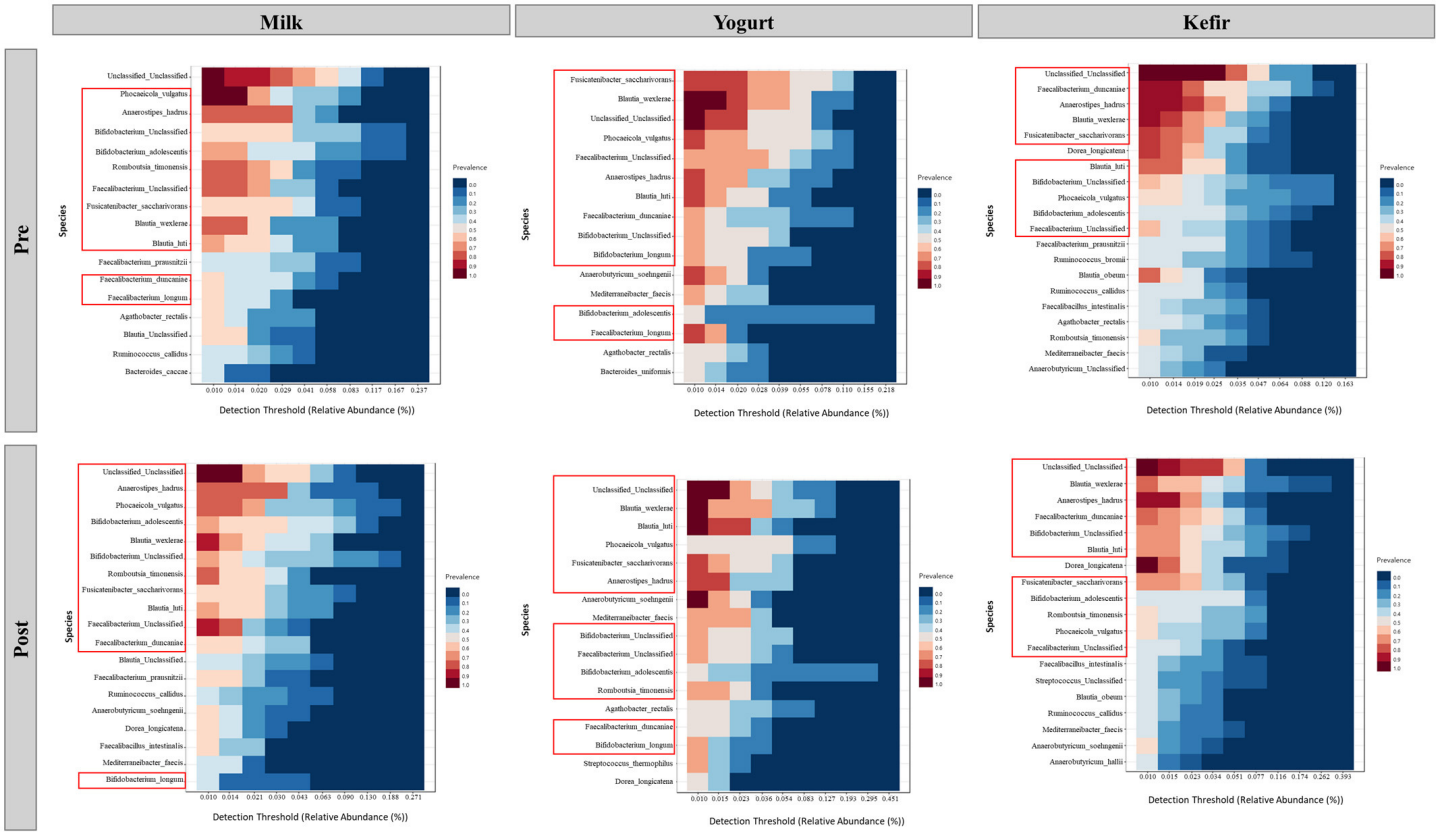
Core microbiome analysis before and after the consumption of milk, yogurt, and kefir. Each panel represents the detection threshold (relative abundance, %) and prevalence of microbial species across samples. The X-axis shows the detection threshold, indicating the relative abundance (%) of each species within the samples, while the Y-axis lists the microbial species identified as part of the core microbiome. Colors represent prevalence, with red shades indicating high prevalence (species found in most samples) and blue shades indicating low prevalence (species found in fewer samples). Red boxes highlight key genera previously identified in the core microbiome.

In this study, we investigated changes in the gut microbiota before and after the consumption of milk, yogurt, or kefir. Species from the genera *Bifidobacterium, Blautia*, and *Faecalibacterium* were identified as part of the dominant core microbiota in all groups. Specifically, *Bifidobacterium adolescentis* and unclassified *Bifidobacterium* species; *Blautia wexlerae* and *Blautia luti*; as well as *Faecalibacterium duncaniae* and unclassified *Faecalibacterium* species consistently maintained a relative abundance of at least 1% in more than 40% of the samples (Figure 4).

### 3.5 Correlation analysis

The SparCC algorithm was applied with a *p*-value threshold of 0.05 and a correlation threshold of 0.4 to identify significant correlations within the gut microbiome, where species abundance relationships are interdependent. Notably, significant correlations were observed among bacterial taxa that increased following supplementation with milk, yogurt, or kefir.

In the milk group, bacteria associated with SCFA production, including *Blautia, Phocaecola*, and *Anaerobutyricum*, exhibited positive correlations with each other. In contrast, *Bifidobacterium* showed negative correlations with these taxa (Table 3). In the yogurt group, *Bacteroides cellulosilyticus* and *Bacteroides xylanisolvens* known for their roles in carbohydrate degradation, displayed a positive correlation with *Blautia luti* (correlation coefficients of 0.97 and 0.54, respectively). Conversely, *Streptococcus* spp., an essential componenet of yogurt fermentation, showed a negative correlation with *Anaerobutyricum soehngenii* (−0.6) and *Mediterraneibacter faecis* (−0.65) (Table 4). In the kefir group, *Bifidobacterium breve* showed a positive correlation with *Akkermensia muciniphila* (0.45), *Ruthenibacterium lactiformans* (0.42), and *Weissella koreensis* (0.55). *Weissella koreensis* demonstrated a strong positive correlation with *Drancourtella massiliensis* (0.72) and *Ruthenibacterium lactiformans* (0.73), but a negative correlation with *Anaerobutyricum soehngenii* (−0.43). *Ruthenibacterium lactiformans* had a positive correlation with *Drancourtella massiliensis* (0.60), while *Faecalibacterium longum* showed a negative correlation with *Anaerostipes hadrus* (−0.46) (Table 5).

**Table 3:** Correlation coefficients of gut microbiome species following milk supplementation.

**Taxon1**	**Taxon2**	**Correlation coefficient**
*Blautia luti*	*Agathobacter rectalis*	0.60
	*Bacteroides uniformis*	−0.55
	*Collinsella aerofaciens*	0.74
	*Mediterraneibacter torques*	−0.55
	*Mediterraneibacter* Unclassified	0.41
*Bacteroides uniformis*	*Anaerobutyricum soehngenii*	0.46
	*Blautia luti*	−0.55
*Anaerobutyricum soehngenii*	*Anaerobutyricum* Unclassified	−0.53
	*Bacteroides uniformis*	0.46
	*Bifidobacterium adolescentis*	−0.58
	*Bifidobacterium* Unclassified	0.66
	*Blautia wexlerae*	0.53
*Blautia wexlerae*	*Agathobacter rectalis*	0.49
	*Alistipes putredinis*	0.53
	*Anaerobutyricum soehngenii*	0.53
	*Bifidobacterium adolescentis*	−0.86
	*Bifidobacterium* Unclassified	0.67
	*Dialister hominis*	0.64
	*Intestinibacter bartlettii*	−0.51
	*Odoribacter splanchnicus*	0.54
*Bifidobacterium adolescentis*	*Alistipes putredinis*	−0.54
	*Anaerobutyricum soehngenii*	−0.58
	*Bacteroides caccae*	−0.43
	*Bifidobacterium* Unclassified	−0.78
	*Blautia wexlerae*	−0.86
	*Clostridium sensu stricto* Unclassified	0.49
	*Dialister hominis*	−0.51
	*Faecalibacterium prausnitzii*	0.48
	*Intestinibacter bartlettii*	0.51
	*Odoribacter splanchnicus*	−0.61
	*Phocaeicola dorei*	−0.74
*Phocaeicola dorei*	*Agathobacter rectalis*	0.66
	*Alistipes putredinis*	0.75
	*Anaerobutyricum soehngenii*	0.44
	*Bifidobacterium adolescentis*	−0.74
	*Bifidobacterium* Unclassified	0.48
	*Blautia wexlerae*	0.77
	*Dialister hominis*	0.5
	*Intestinibacter bartlettii*	−0.53

**Table 4:** Correlation coefficients of gut microbiome species following yogurt supplementation.

**Taxon1**	**Taxon2**	**Correlation coefficient**
*Agathobacter rectalis*	*Bacteroides cellulosilyticus*	0.98
	*Blautia luti*	0.98
	*Faecalibacterium duncaniae*	0.77
*Blautia luti*	*Agathobacter rectalis*	0.98
	*Bacteroides cellulosilyticus*	0.97
	*Bacteroides xylanisolvens*	0.55
	*Faecalibacterium duncaniae*	0.77
*Faecalibacterium duncaniae*	*Agathobacter rectalis*	0.77
	*Bacteroides cellulosilyticus*	0.75
	*Blautia luti*	0.77
	*Phocaeicola vulgatus*	−0.52
*Bacteroides cellulosilyticus*	*Agathobacter rectalis*	0.98
	*Blautia luti*	0.97
	*Faecalibacterium duncaniae*	0.75
*Bacteroides xylanisolvens*	*Bifidobacterium* Unclassified	−0.58
	*Blautia luti*	0.55
	*Mediterraneibacter faecis*	0.63
*Bifidobacterium adolescentis*	*Bifidobacterium longum*	0.66
	*Bifidobacterium* Unclassified	0.67
	*Faecalibacterium* Unclassified	0.67
	*Fusicatenibacter saccharivorans*	0.98
*Bifidobacterium longum*	*Alistipes onderdonkii*	0.64
	*Anaerostipes hadrus*	−0.69
	*Bifidobacterium adolescentis*	0.66
	*Fusicatenibacter saccharivorans*	0.67
	*Odoribacter splanchnicus*	0.85
*Fusicatenibacter saccharivorans*	*Bifidobacterium adolescentis*	0.98
	*Bifidobacterium longum*	0.67
	*Bifidobacterium* Unclassified	0.65
	*Faecalibacterium* Unclassified	0.65
	*Phocaeicola dorei*	−0.57
*Streptococcus* Unclassified	*Anaerobutyricum soehngenii*	−0.62
	*Mediterraneibacter torques*	−0.65

**Table 5:** Correlation coefficients of gut microbiome species following kefir supplementation.

**Taxon1**	**Taxon2**	**Correlation coefficient**
*Anaerobacterium chartisolvens*	*Bilophila wadsworthia*	0.44
	*Roseburia intestinalis*	0.42
*Bifidobacterium breve*	*Akkermansia muciniphila*	0.45
	*Anthropogastromicrobium aceti*	0.53
	*Parasutterella excrementihominis*	0.41
	*Ruthenibacterium lactatiformans*	0.42
	*Weissella koreensis*	0.55
*Pararoseburia lenta*	*Anaerobulyricum soehngenii*	−0.44
	*Faecalibacillus faecis*	0.47
	*Faecalicatena Unclassified*	0.43
	*Muricoprocola aceti*	0.43
	*Roseburia amylophila*	0.41
*Lactococcus lactis*	*Alistipes onderdonkii*	0.47
	*Alistipes putredinis*	0.42
	*Parasutterella excrementihominis*	0.41
*Weissella confusa*	*Enterocloster Unclassified*	0.45
	*Senegalimassilia anaerobia*	0.41
*Weissella koreensis*	*Anaerobutyricum soehngenii*	−0.43
	*Coprobacter secundus*	0.49
	*Drancourtella massiliensis*	0.72
	*Roseburia amylophila*	0.47
	*Ruthenibacterium lactatiformans*	0.73
*Ruthenibacterium lactatiformans*	*Bacteroides fragilis*	0.42
	*Blautia faecis*	0.47
	*Coprobacter secundus*	0.41
	*Drancourtella massiliensis*	0.6
	*Flavonifractor plautii*	0.55
	*Thomasclavelia ramosa*	0.42
	*Thomasclavelia spiroformis*	0.51
*Anaerotignum faecicola*	*Oscillibacter acetigenes*	0.47
	*Paraprevotella clara*	0.51
	*Streptococcus Unclassified*	−0.57
	*Waltera intestinalis*	0.45
*Blautia luti*	*Mediterraneibacter faecis*	0.41
*Faecalibacterium longum*	*Anaerostipes hadrus*	−0.46
	*Dialister hominis*	0.45
	*Thomasclavelia ramosa*	0.51

### 3.6 Predicted functional pathway

**Figure 5:**
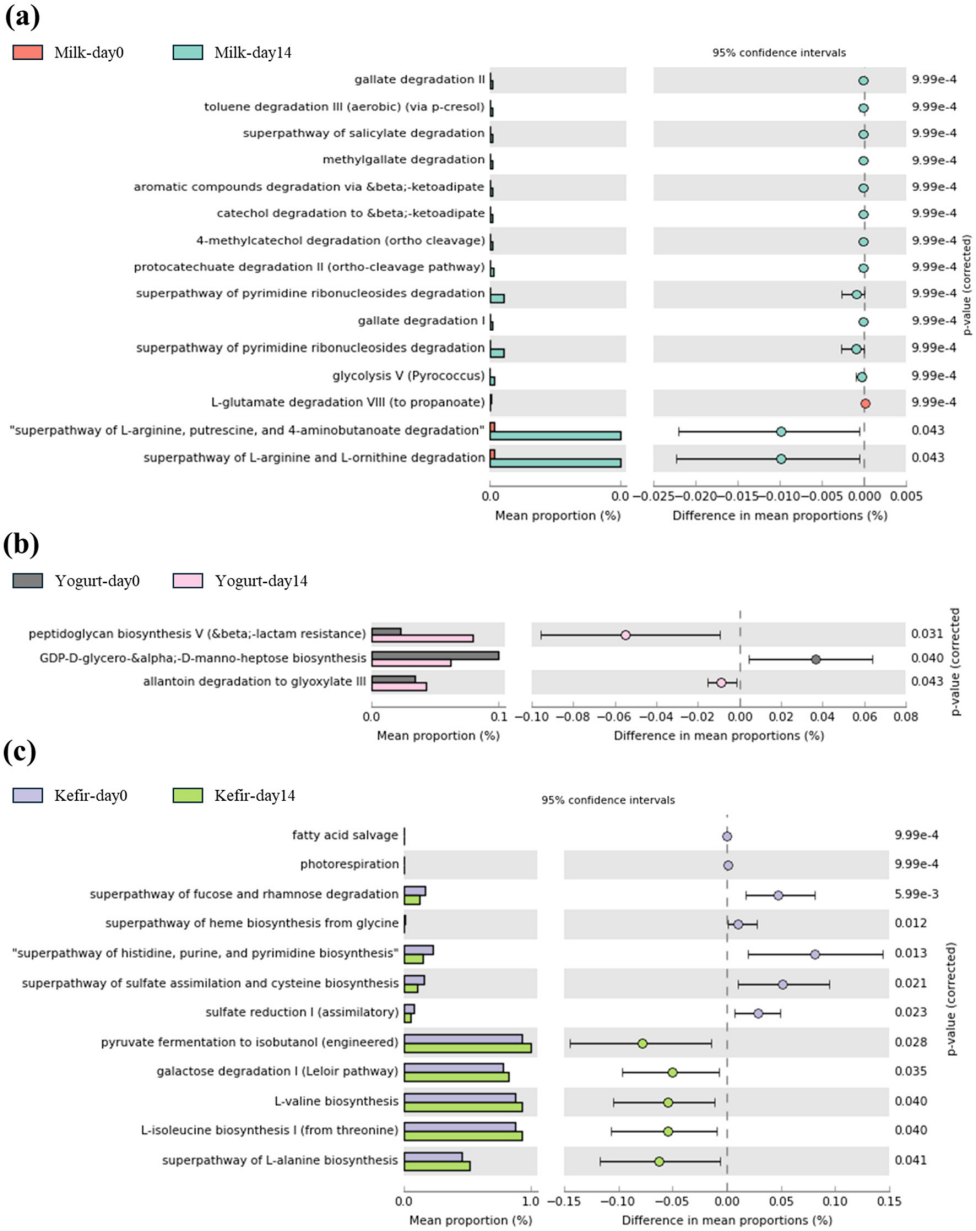
Comparison of predicted metabolic pathway abundances before and after consuming **(a)** milk, **(b)** yogurt, and **(c)** kefir using PICRUSt2 with MetaCYC database.

We performed PICRUSt2 analysis based on the MetaCYC database to predict functional changes in microbial metabolic gene pathways within the gut microbiome. In the milk group, metabolic pathways associated with the degradation of aromatic compounds (0.00%−0.03%), including gallate (0.00%−0.03%), catechol (0.00%−0.03%), pyrimidine (0.00%−0.04%), were significantly enriched after the intervention (*p* < 0.05) (Figure 5a). In the yogurt group, increased activity was observed in the peptidoglycan biosynthesis V (0.06%−0.08%) and allantoin degradation to glyoxylate III pathways (0.02–0.03%), suggesting enhanced bacteria cell wall biosynthesis and nitrogen metabolism (*p* < 0.05). In contrast, GDP-D-glycero- α-D-manno-heptose biosynthesis, which is associated with lipopolysaccharides (LPS), components of the outer membrane of gram negative bacteria, showed a significant decrease (0.08%−0.06%) (*p* < 0.05) (Figure 5b). After kefir supplementation, a significant increase in the abundance of the pyruvate fermentation to isobutanol (0.9%−1.0%), galactose degradation (Leloir pathway) pathways (0.7%−0.9%) (*p* < 0.05), and pathways related to proteinogenic amino acid biosynthesis were observed. The mean relative abundance of these pathways increased significantly from baseline to day 14 (*p* < 0.05) (Figure 5c). These functional changes were specific to each group and were not observed across all treatment groups.

## 4 Discussion

In this study, healthy young adults participated in a two-week residential camp where they consumed standardized meals and shared the same living environment. This design minimized dietary and environmental variability, providing a controlled setting to assess the effects of fermented milk products on the gut microbiota. This randomized controlled trial demonstrated that kefir consumption altered gut microbiota composition. Notably, kefir intake increased the relative abundance of key lactate-producing bacteria, such as *Bifidobacterium, Ruthenibacterium, Weissella*, and *Leuconostoc*. Furthermore, species within the genus *Blautia*, known for their role in SCFA production, also increased following kefir intake. These microbial shifts suggest that kefir has the potential to beneficially modulate the gut microbiota by promoting the growth of health-associated bacteria. Such changes may contribute to improved gut health and support overall host wellbeing.

Core microbiome analysis before and after the consumption of milk, yogurt, or kefir revealed that species within the genera *Bifidobacterium, Blautia*, and *Faecalibacterium* remained dominant, consistently maintaining their relative abundance. The stability of these core microbiota suggests that while dietary interventions may induce temporary changes in specific microbial taxa, the core microbiome exhibits notable resilience. This resilience reflects a well-adapted gut ecosystem in which essential microbial functions are preserved despite dietary changes. The persistence of core gut microbiota highlights their crucial roles in sustaining gut health by sustaining essential microbial functions in the face of external influences (Fassarella et al., 2021).

At the genus level, notable changes were observed in *Bluatia*, a genus known for producing acetate and propionate, that promote mucus secretion and support gut barrier integrity (Holmberg et al., 2024). Specifically, there was a significant increase in *Blautia wexlerae* and *Blautia luti* in the kefir group. Additionally, kefir consumption led to increased levels of lactate-producing bacteria such as *Bifidobacterium breve, Ruthenibacterium lactatiformans, Weissella koreensis*, and *Leuconostoc mesenteroides*. These bacteria are particularly important due to their association with SCFAs production (Fusco et al., 2023; Keum et al., 2024). SCFAs, including acetate, butyrate, and propionate, are the primary metabolic products produced by the gut microbiota during the fermentation of undigested polysaccharides (Martin-Gallausiaux et al., 2021; Zhang et al., 2020). These metabolites play critical roles in enhancing the intestinal barrier, facilitating nutrient absorption, and maintaining gut homeostasis (Tan et al., 2014; Ducarmon et al., 2019).

*Bifidobacterium breve*, an anaerobic bacterium commonly found in the gut of healthy infants, plays a crucial role in gut health by converting lactose into acetate, ethanol, and formate, thereby generating energy and contributing to an acidic intestinal environment (Bozzi Cionci et al., 2018; Camargo et al., 2023). Similarly, *Weissella koreensis*, a key lactic acid bacterium involved in kimchi fermentation, uses a heterofermentative pathway to convert carbohydrates into lactate and acetate (Ahmed et al., 2022). These metabolic activities contribute to acetate production that plays a vital role in maintaining an acidic environment and inhibiting the growth of harmful bacteria, thereby supporting overall gut health (Fukuda et al., 2011). The fermentation of *Leuconostoc mesenteroides* is associated with butyrate production and is known to stimulate the growth of *Bifidobacterium* through the production of 1,4-dihydroxy-2-naphthoic acid (DHNA) (Traisaeng et al., 2020). *Ruthenibacterium lactatiformans* participates in butyrate-related pathways by fermenting lactate and succinate, which serve as substrates for other microbial taxa that directly produce butyrate (Becker et al., 2022).

In the correlation analysis, *Bifidobacterium breve, Ruthenibacterium lactiformans*, and *Weissella koreensis* showed positive correlations with one another. *Akkermensia muciniphila*, which was positively correlated with *Bifidobacterium breve*, is known for its ability to degrade mucin, thereby supporting mucus layer regeneration and maintaining gut barrier integrity (Derrien et al., 2017). Similarly, gut microbiota commonly isolated from the feces of healthy individuals also showed positive correlations. *Drancourtella massiliensis* showed positive correlations with both *Weissella koreensis* and *Ruthenibacterium lactiformans* (Durand et al., 2016). These positive correlations among gut health-associated species suggest the presence of potential mutualistic relationships or a shared preference for specific gut environmental conditions.

In contrast, negative correlations were observed between *Anaerobutyricum soehngenii* and *Weissella koreensis*, as well as between *Anaerostipes hadrus* and *Faecalibacterium longum*. Both *Anaerobutyricum soehngenii* and *Anaerostipes hadrus* are known butyrate producers (Liu et al., 2024; Wortelboer et al., 2022). These negative correlations may indicate competitive interactions within the gut microbial community, potentially related to substrate utilization. Overall, the observed microbial interactions reflect the complexity of a balanced microbial ecosystem that is capable of adapting to dietary changes while preserving gut health and functional stability (Fassarella et al., 2021; Bäckhed et al., 2015).

In the case of *Streptococcus thermophilus*, a bacterium commonly used in yogurt fermentation, an increase in abundance was observed only in the yogurt group. In contrast, its abundance decreased in both the milk and kefir groups. This decline is likely attributable to the fact that 5 out of 9 participants in the milk group and 9 out of 13 participants in the kefir group had regularly consumed yogurt prior to the study, but discontinued its intake during the intervention period, resulting in a reduction in *S. thermophilus* abundance.

PICRUSt2 analysis revealed increased activity in the Leloir pathway for galactose degradation following kefir consumption. Kefir contains *Lactobacillus kefiranofaciens* and *Lentilactobacillus kefiri*, which possess enzymes such as β-galactosidase that hydrolyze lactose into simpler sugars including galactose and glucose (He et al., 2021). As fermentation progresses, yeasts such as *Kazakhstania unispora* and *Dekkera anomala* further contribute to lactose breakdown, increasing galactose levels. Kefir fermentation has been shown to elevate levels of certain metabolites, including galactose (Tingirikari et al., 2024). Once released, galactose is first converted into glucose-1-phosphate, then into glucose-6-phosphate, which enters glycolysis to produce pyruvate (Koh et al., 2016).

In summary, this study investigated the effects of kefir-fermented milk consumption on gut microbial composition in healthy young adults, under controlled dietary and environmental conditions. By minimizing individual dietary variability, this study adds valuable insight to human gut microbiome research.

However, this study has several limitations. Functional predictions were made using PICRUSt2, an indirect method that infers microbial metabolic pathways based on 16S rRNA data. These predictions should be interpreted with caution and ideally validated using direct methods such as metabolomics or transcriptomics. In addition, the relatively small sample size, due to practical constraints, has limited the power to detect small to moderate effects. Larger studies are necessary to confirm these findings and to improve statistical robustness.

Moreover, the short duration of the intervention limits understanding of the long-term effects of kefir consumption on the gut microbiome. Future studies should include longer follow-up periods to assess the persistence of observed changes. Also, the lack of blinding in outcome assessments in this study presents a potential source of bias. Future trials would benefit from incorporating blinded assessments to minimize this risk.

Although all participants received the same meals during the study period, actual food intake was not monitored. Including food diaries or 24-h dietary recall in future studies would help ensure compliance and offer additional context for interpreting microbiome changes.

## Data Availability

The data presented in the study are deposited in the NCBI repository, accession number PRJNA1212986.

## Supplementary material

Supplementary Figure 1 Baseline beta-diversity across the three groups before the intervention.

Supplementary Table 1 Quantitative data of microbial taxa corresponding to Figure 2.
