# Supplementary figures and images for: Evaluation of kefir consumption on gut microbial diversity in a healthy young population using full-length 16S rRNA sequencing
Source: Front Microbiol. 2025 May 21;16:1587831. doi: 10.3389/fmicb.2025.1587831 (PMC12134070; doi:10.3389/fmicb.2025.1587831)

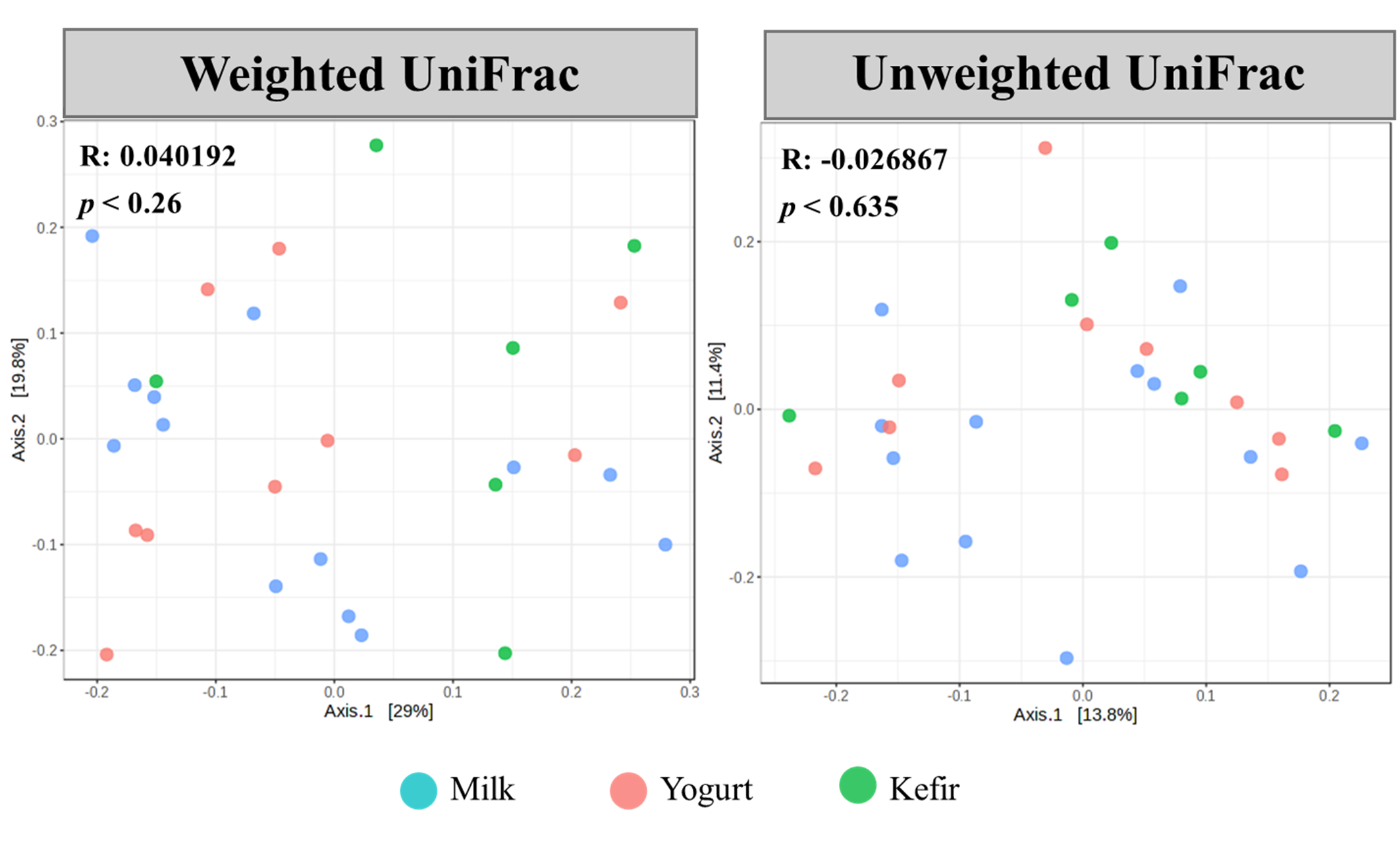

Supplement: Supplementary Figure 1 — Baseline beta-diversity across the three groups before the intervention. [file Image_1.tif]
